# Mechanical Performance of Direct Restorative Techniques Utilizing Long Fibers for “Horizontal Splinting” to Reinforce Deep MOD Cavities—An Updated Literature Review

**DOI:** 10.3390/polym14071438

**Published:** 2022-04-01

**Authors:** András Jakab, András Volom, Tekla Sáry, Eszter Vincze-Bandi, Gábor Braunitzer, David Alleman, Sufyan Garoushi, Márk Fráter

**Affiliations:** 1 Department of Operative and Esthetic Dentistry, Faculty of Dentistry, University of Szeged, H-6720 Szeged, Hungary; jakab.andras.gabor@gmail.com (A.J.); teklasary@gmail.com (T.S.); vbeszter96@gmail.com (E.V.-B.); 2 Volom Dental, H-1037 Budapest, Hungary; drvolom@drvolomdental.hu; 3 dicomLAB Dental Ltd., H-6726 Szeged, Hungary; braunitzergabor@gmail.com; 4 The Alleman Center for Biomimetic Dentistry, South Jordan, UT 84095, USA; allemancenter@gmail.com; 5 Department of Biomaterials Science and Turku Clinical Biomaterials Center-TCBC, Institute of Dentistry, University of Turku, FI-20520 Turku, Finland; sufgar@utu.fi

**Keywords:** fiber reinforcement, long fibers, polyethylene fiber, fracture resistance, fracture pattern, horizontal splinting, transcoronal fixation

## Abstract

Excessive cavity preparation and root canal treatment leads to a weakened tooth structure with a lower resistance to fracture. Fiber reinforcement is frequently used to reinforce such teeth, and multiple fiber types and possible applications exist. Various methods for utilizing long fibers to internally splint the remaining cavity walls in the case of large mesio-occluso-distal (MOD) cavities have been proposed; however, no summary of their performance has been written up to now. Our study aims to review the available literature to evaluate and compare the mechanical performance of the different materials and methods utilized for horizontal splinting in large MOD cavities. Three independent authors performed a thorough literature search using PubMed, ScienceDirect, and Google Scholar up until January 2022. The authors selected in vitro studies that used long fibers placed horizontally in posterior teeth with large MOD cavities to reinforce these teeth. From 1683 potentially relevant articles, 11 publications met our inclusion criteria. Seven out of eleven studies showed that horizontal splinting with long fibers improved the fracture resistance of the restored teeth. Three articles showed no significant difference between the fracture resistance of the restored groups. Only one article reported a lower fracture resistance to the horizontally splinted group, compared to conventional direct composite restoration. Within the limitations of this review, evidence suggests that long fiber reinforcement could be used to improve the fracture resistance of heavily restored teeth.

## 1. Introduction

The restoration of root-canal-treated (RCT) teeth is one of the most challenging tasks in dentistry. Excessive cavity preparation and root canal treatment leads to the loss of a great amount of tooth material. Due to the reduction in tooth structure, these teeth have a lower resistance to fracture [1]. In upper premolars, the loss of one marginal ridge leads to a 46% loss in tooth rigidity, while an MOD preparation results in an average decrease of 63% in cuspal stiffness [2]. Apart from the mechanical properties, the marginal integrity of these restorations is also very important. The seal of the coronal restoration of an endodontically treated tooth has a great impact on the success of the root canal treatment and, for the above mentioned reasons, the restoration of RCT teeth is a frequently discussed question among clinicians [3].

The need for tooth structure reinforcement in such cases had been in focus for a couple of years. There have been numerous suggestions in the past years for the long-lasting restoration for such teeth; however, no consensus has been reached. Fiber-reinforced composite (FRC) posts have been used to restore RCT teeth in the past decades to increase the retention of the core build-up material [4]. The results of studies investigating the possible tooth-strengthening effect of the conventional FRC posts have been contradictory throughout the years. Several studies reported that the use of FRC posts increased the fracture resistance of RCT premolars [5,6]. In contrast, other researchers suggested that the use of FRC posts did not increase the fracture resistance of the restored teeth, and even reported the possible weakening of the root due to the post space preparation [7,8]. 

Resin composite materials are widely used to restore MOD cavities. Composite materials can bond to tooth surfaces and can act as a splint between the remaining tooth structures. However, there are some limitations of composite materials that can influence the long-term success of large direct restorations. Conventional resin composites have a significantly lower fracture toughness compared to dentine [9]. Furthermore, the polymerization shrinkage-related stress increases with the cavity depth due to an increase in the C-factor (ratio of bonded and unbonded surfaces in the cavity) and volume-factor (size, mainly the depth of the cavity), leading to greater stress on the cavity walls. This taking place at the restoration–tooth interface can manifest in micro-leakage, secondary caries, and even the fracture of the remaining cavity walls [10]. For the mentioned reasons, the reinforcing capabilities of resin composites are highly debated [11]. A large MOD preparation usually leaves thin buccal and lingual walls, which are prone to fracture. Some studies suggest that it is necessary to perform cuspal coverage for deep MOD cavities to prevent the fracture of the remaining walls [12,13]. 

In recent years, many different innovative restoration techniques and new materials have appeared, utilizing fiber reinforcement. The use of fibers in dentistry has expanded the possible applications of direct restorations, as they are capable of reinforcing the restoration [14]. Short fiber-reinforced composite (SFRC) materials are a good option for dentine replacement in extensive preparations, as they can act as a stress-absorbing layer in the restoration [15]. In SFRC materials, the fibers are randomly oriented, and reinforcement occurs in three directions. In contrast, bidirectional and woven continuous fibers provide reinforcement in only two directions; however, this reinforcement is stronger than it is in SFRC materials. Bidirectional FRC (e.g., EverStick Net; GC Europe, Leuven, Belgium) and leno woven ultra-high molecular weight (LWUHMW) polyethylene fiber ribbon (Ribbond THM; Ribbond Inc., Seattle, WA, USA) have been used in various direct restorative techniques. Apart from the capability of acting as a stress-absorbing layer in the restoration, these fibers are suggested to act as an internal splint to increase the fracture resistance [16,17]. 

**Figure 1:**
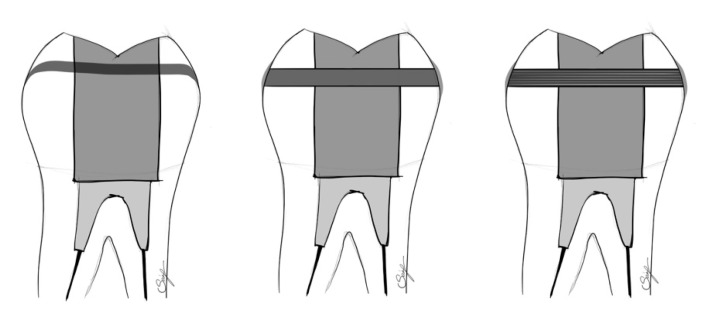
Schematic figure of occlusal splinting with polyethylene or long glass fibers (**left**), of transcoronal fixation with polyethylene fibers (**middle**), and of horizontally positioned glass fiber post (**right**).

The transcoronal splinting of the remaining buccal and lingual walls of a tooth with an MOD cavity is a technique described in recent publications. Long fibers (such as FRC posts, continuous bidirectional FRCs, and polyethylene fibers) are utilized to internally splint the cavity walls to increase the fracture resistance of the tooth. The fibers are placed horizontally, either through a small hole prepared on the buccal and lingual walls or in a groove drilled in the occlusal surface of the restoration. A schematic figure of the most commonly used horizontal splinting techniques is shown in Figure 1.

The question arises whether this technique could be used to strengthen teeth with large MOD cavities. Our study aims to collect and evaluate the available evidence on this subject. 

## 2. Materials and Methods

### 2.1. Search Strategy

Sources: A literature review was carried out with the use of PubMed, ScienceDirect, and Google Scholar up until January 2022. The following keywords were used to collect articles: “polyethylene fiber”, “horizontal glass fiber”, “horizontal fiberglass”, “fiber-reinforced restoration”, “Ribbond”, “glass fiber post”, “fiber reinforcement”. The search focused on articles mainly from the last 10 years; however, some older papers were also included due to their high relevance. After removing the duplicates, three authors carefully reviewed the titles and abstracts of the publications. A title was discarded if all three authors agreed that it was irrelevant to the review. Eligible abstracts were retained for full-text review [18].

### 2.2. Eligibility Criteria

Eligible studies for inclusion are full-text in vitro studies that used long fibers placed horizontally in large MOD cavities to restore the remaining tooth structure. Only studies testing fracture toughness, fracture strength, fracture resistance, and failure mode were included in this review. The included studies are published in peer-reviewed journals, in English language, and the search terms were included in either the title or the abstract. All studies used extracted human molars and premolars.

### 2.3. Data Synthesis

The included studies were carefully read and the relevant information was collected in a Microsoft Word document. The following details were recorded for each included publication: authors’ names, the title of the article, year of publication, experimental groups, presence of control group, the type of long-fiber used, type of application of long-fibers, the main results, and conclusions.

### 2.4. Quality Assessment

Risk of bias was determined for each article by three authors, independently. The following parameters were used to determine the risk of bias, according to previous systematic reviews: the presence of a control group; sample size calculation; standardization of the preparation of the samples; sample randomization; sample preparation by a single operator; blindness of the operator; failure mode evaluation [18,19]. For each article, the amount of these parameters that were mentioned in the article was counted. The risk of bias was determined as follows: high risk (1–2 parameters), medium risk (3–5 parameters), low risk (6–7 parameters).

## 3. Results

**Figure 2:**
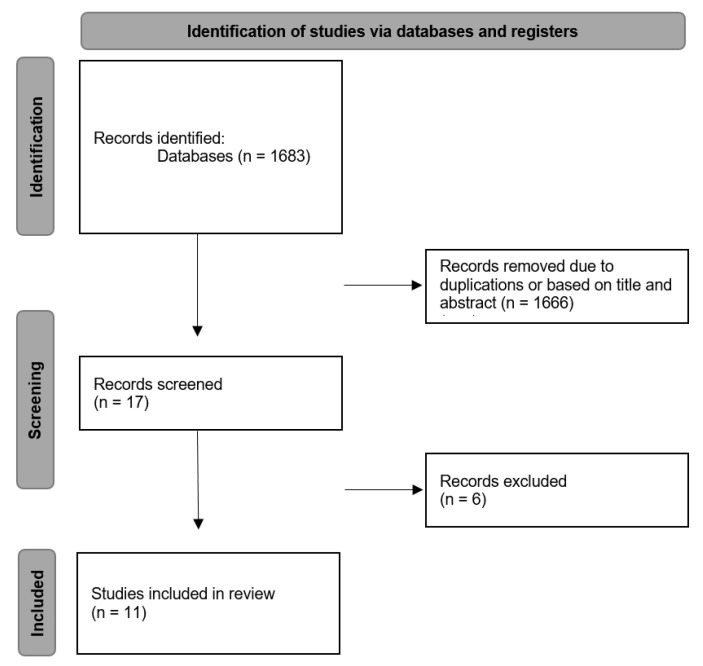
PRISMA flow diagram of the screening and selection process.

A total of 1683 relevant articles were recognized and screened through title and abstract evaluation. After careful assessment, 1666 articles were removed because they did not meet the inclusion criteria or were duplicates. Thus, 17 articles were selected based on their relevance to this review. After screening the full texts, six further studies were excluded because they reported on a single clinical case only, or they covered only finite element analyses. This left us with 11 articles for the full-text analysis. Figure 2 shows the screening and selection process in a PRISMA flow diagram [18]. 

Ten out of eleven studies used RCT molars and premolars with class II MOD cavities, whereas one study used non-endodontically treated molars [20]. Seven studies tested glass fibers as horizontal reinforcement and three articles tested polyethylene fibers. All included publications investigated the fracture strength of the restorations. One study compared a pre-impregnated fiberglass net with polyethylene fiber net [20]. Seven publications reported an increased fracture resistance for the horizontally splinted groups [20,21,22,23,24,25]. From the other four studies, three reported no significant difference for the fracture resistance of the horizontal long-fiber reinforcement, compared to direct composite restorations [26,27]. Only one article stated that the horizontal intercuspal splinting resulted in a lower fracture resistance compared to the conventional composite restoration [28]. Regarding the fracture pattern, five out of the included studies reported a positive effect of long-fiber reinforcement on the fracture pattern [22,24,26,27,29]. On the other hand, four articles reported less favorable fracture patterns in the horizontally splinted groups [21,25,30], whilst two articles provided an inadequate amount of information on this matter [23,28]. Table 1 summarizes the details of the included publications.

Table 2 summarizes the risk of bias assessment. All included studies showed a medium risk. The operator blindness, sample size calculation, and single operator were missing from most of the publications.

Table 3 summarizes the testing methodology within the reviewed articles.

## 4. Discussion

Fiber reinforcement is a frequently discussed subject among researchers and clinicians. The need for strengthening the tooth structure after excessive preparation is part of the everyday dental routine. Endodontic treatments, the replacement of large direct amalgam fillings, or large decays often lead to large cavities with weakened remaining walls [1]. With the development of adhesive technology and the appearance of strong composite materials, large MOD cavities in molars and premolars are routinely restored with direct composite fillings [31]. However, there are some limitations with composite fillings that need to be addressed during the restoration of such cavities. One of the problems with direct composite fillings is polymerization shrinkage, which can lead to micro-leakage and recurrent caries [2]. This can partly be addressed by the incremental layering or the decoupling-with-time concept [32]. Another problem with composite restorations is their inadequate fracture toughness. Modern composites are rigid, strong materials, but they lack fracture toughness, which is the resistance to the propagation of cracks under loading [33]. As a result of these limitations, direct composite restorations might not be the best solution for excessive MOD cavities in posterior teeth [20]. Fiber reinforcement in composite restorations tends to strengthen the restoration and the structurally compromised tooth [14]. The size, type, and orientation of the fibers could all be significant factors in the potential strengthening effect of these materials. In SFRC materials, the fibers are randomly oriented and provide some strengthening in all directions. Bidirectional and woven fibers are oriented in two directions; however, their strengthening effect is stronger in those directions compared to the SFRC [34]. Furthermore, these long fibers can act as an internal splint that connects the remaining tooth structure [16,17]. Another topic that needs to be discussed is the fracture pattern of restored teeth. Teeth with large MOD cavities have limited tooth structure left. It is important that, if the restoration fails, it should fail in a way where the tooth remains restorable. Fibers have shown the ability to re-direct and/or stop crack propagation in composite restorations [35]. Numerous approaches have been suggested to place fibers inside direct restoration. However, not all solutions are suitable to reinforce deep MOD cavities [20]. SFRCs are easy-to-use and provide a time-efficient option to replace dentine. However, the randomly oriented fibers might not result in the strongest reinforcement that we could achieve. When long fibers are used to stabilize the opposing walls, they can not only act as an internal splint, but also as a potential stress-absorbing layer [16,17]. With the well-defined orientation of long fibers, the exact placement of the fibers may become important. Sáry et al. showed that, whenever polyethylene fibers are used in an MOD cavity, irrespective of their position, as long as the remaining walls were connected, the fracture resistance was improved (2129–2484 ± 629–682 N) compared to composite fillings without fibers being incorporated (1629 ± 503 N) [20]. However, this was not the case when the FRC net (everStick NET, GC Europe, Leuven) was used in the same setup [20]. It is most likely that this difference can be traced back to the difference between the characteristics of the two types of fibers. The FRC net contains bidirectional glass fibers, providing orthotropic properties to the material [34]. As the material is slightly more rigid from a handling point of view compared to polyethylene fibers, it may not be perfectly adaptable to an uneven (cavity) surface, which can lead to a gap formation between the net and the bonded surface. Other FRC nets could be used for fixation or internal connection in cavities and/or restorations. Daher et al. [22] and Küçük et al. [24] used an FRC pre-impregnated strip (Dentapreg) to splint the remaining buccal and lingual walls. Dentapreg fibers are based on the S2 glass system embedded in Bis-GMA and TEGDMA in a cross-linked polymer matrix. They contain 8300 unidirectional fibers coated with plasma-enhanced chemical vapor deposition [36]. Polyethylene fibers are characterized by a dense concentration of fixed nodal intersections, which aids the maintenance of the integrity of the fabric. This enables the stresses in the bulk of the material to be transferred more effectively because of the well-defined load paths from one area to another [27]. According to Rudo and Karbhari, the favorable performance of the polyethylene fibers is due to the unique properties of the fiber, the chemical bonding between the fiber and the resin, and the effect of the leno weave with regard to crack resistance and deflection, as well as the resistance to shifting within the matrix [16]. The intracoronal splinting of the opposing walls can also be performed by using conventional FRC posts inserted through artificial holes in the remaining buccal and lingual walls of the cavity. Authors promoting this technique emphasize that the method is cost-effective (compared to indirect techniques) and that it is a simple way to reinforce the dental structures after root canal treatment [37]. However, it is the most invasive method among the horizontal splinting techniques.

These approaches all aim to reinforce large and deep MOD cavities by stabilizing the remaining structures. Placing long fibers transcoronally/horizontally within direct restorations is not only time-efficient and more available to all patients (being less costly), but could also hold the potential to replace indirect cuspal coverage restorations in these cases. However, there is no scientific consensus on the mechanical performance of these restorations in this topic, and this is the reason why we sought to gather all available in vitro evidence in this review. Based on the selected studies, the most frequently tested parameters were the fracture resistance and fracture pattern.

### 4.1. Fracture Resistance

The fracture resistance of restored teeth is highly dependent on the amount of remaining tooth structure [1]. To enhance the strength of these teeth and their restorations, different materials and different methods of application have been introduced. The horizontal placement of long fibers could be a potential method used to reinforce weakened posterior teeth.

Seven of the included studies reported an increased fracture resistance of teeth restored with different long fibers. Three studies tested polyethylene fibers (Ribbond) for horizontal splinting utilizing the occlusal splinting method. In the study of Belli et al. [23] and Sáry et al. [20], polyethylene fibers applied this way exhibited an improved fracture resistance (1224 ± 132 N, 2129–2484 ± 629–682 N) compared to teeth restored with composite filling without fibers (749 ± 124 N, 1629 ± 503 N). This is in accordance with previous research showing that polyethylene fibers incorporated into composite filling enhances the filling’s mechanical performance [17]. However, these results contradict the findings of Akman et al., who found that the placement of polyethylene fibers inside the cavity (including the occlusal splinting method), did not result in restorations with a significantly higher fracture resistance (1853 ± 297 N) compared to composite fillings (1798 ± 180 N) [27]. This might be attributed to the difference in their study setups. The speed of the load to fracture testing in the study of the Akham group was 5 mm/min, which is much higher than the generally applied 0.5–2 mm/min [38,39].

Bahari et al. [26] and Küçük et al. [24] used the same restorative method (i.e., occlusal splinting) but with different fibers (Interlig FRC fibers [26], and Dentapreg FRC fibers [24]). Küçük et al. [24] managed to demonstrate an improved fracture resistance (1138 ± 168 N), and the occlusally splinted teeth not only outperformed the ones restored with the composite only (611 ± 194 N), but they did not differ significantly from sound teeth (1190 ± 495 N) either. In contrast, Bahari et al. [26] did not find any improvement when using long fibers for occlusal splinting. This could be due to the difference in the amount of fibers within the reinforcing/splinting materials.

Six studies evaluated horizontal splinting with conventional FRC posts. Scotti et al. [30], Karzoun et al. [25], and Bromberg et al. [21] all managed to show a significantly higher fracture resistance in the case of using horizontal FRC posts (582 ± 76 N, 961 ± 245 N, 2693 ± 372 N) compared to composite filling without FRC posts (364 ± 48 N, 482 ± 72 N, 1680 ± 454 N). One possible explanation for this could be the reduction in cusp deflection caused by anchoring of the buccal and lingual walls of the cavity preparation due to the post insertion [21]. Another benefit of using FRC posts for this technique is their low elastic modulus, which is similar to dentin, leading to an even distribution of the load forces [40]. Furthermore, three of the selected publications reported that the horizontally splinted groups did not differ significantly from teeth restored with cusp-covered overlays [21,22,29]. This suggests that the horizontal application of long fibers could be an alternative treatment to cusp-coverage-indirect restorations. However, more in vitro and preferably in vivo investigations are necessary to support these results.

Sáry and colleagues introduced the transcoronal fixation technique, in which, polyethylene fibers are used to internally splint the opposing walls [20]. In their study, transcoronal fixation showed the highest fracture resistance (2484 ± 682 N) of all tested approaches. Furthermore, it did not differ in terms of the fracture resistance from healthy, intact teeth (2266 ± 601 N). The concept is the same as in splinting with FRC posts, as, in transcoronal fixation, the polyethylene fibers are positioned through artificial holes in the remaining walls. However, this method is different from other known ways of polyethylene fiber application, as, here, fibers are not just placed into the cavity but placed under tension. Supposedly, this way of splinting the remaining walls is more efficient, allowing less cuspal movement. Polyethylene fibers seem ideal for this technique, as these fibers have a very high modulus of elasticity, which results in a resilience to stretch and distortion. Furthermore, their closed-stitch structure provides a very high resistance to traction [41].

These favorable results with horizontal splinting contradict the findings of Mergulhao et al. [29], Bahari et al. [26], and Abou-Elnaga et al. [28], who did not find a significant difference when comparing the fracture resistance of MOD molar cavities restored with composite fillings with (934 ± 233 N, 1023 ± 295 N, 1696 ± 358 N) or without (999 ± 352 N, 1103 ± 378 N, 1723 ± 453 N) horizontal FRC posts. This could be caused by the extreme weakening of the posterior tooth during an MOD situation and root canal treatment. The depth of the cavity preparation, as well as the presence or absence of the marginal ridges, have been shown to be the most critical factors for generating stress in the cavity walls [42]. Cuspal deflection increases with increasing cavity dimensions [43]. Hood reported that the floor of the cavity serves as a fulcrum for cusp bending, and the cantilever length increases with the depth of the cavity [44]. This is in accordance with the findings of Forster et al. [45]. The authors would like to stress that, due to the already mentioned reasons, conventional direct composite fillings are not ideal either to restore or reinforce root-canal-treated MOD cavities. This has been shown several times and is widely accepted [20,45]. Claims to the contrary should be considered with caution. 

Abou-Elnaga and co-workers found that the horizontal application of long fibers (1696 ± 358 N) did not improve the fracture resistance compared to teeth restored with conventional direct composite restorations (1977 ± 316 N) [28]. The authors created an artificial truss-access in endodontically treated mandibular molars, utilizing a glass fiber post transcoronally. Whereas the real truss-access cavities (1723 ± 453 N) did not differ significantly from the intact teeth (2260 ± 540 N), the group with conventional access cavity restored with direct composite filling (1977 ± 316 N) performed worse than the intact teeth (2260 ± 540 N). It must be noted that Abou-Elnaga et al. used only one FRC post to stabilize the remaining walls in molar teeth, whereas the original description of the method recommends two [21]. This could explain the difference.

From a mechanical point of view, most horizontal splinting techniques seem to increase the fracture resistance in the case of deep MOD cavities compared to direct composite restorations without any horizontal fiber reinforcement. So far, none of the studies directly compared horizontal splinting techniques with each other, except for Sáry et al. [20], where there was no difference in the fracture resistance between the occlusal (2129 ± 629 N) and the transcoronal (2484 ± 682 N) splinting, both being performed with the aid of polyethylene fibers. So far, it seems that horizontal splinting can be performed with multiple materials (e.g., polyethylene, FRC post, bidirectional FRC fibers, etc.) in deep MOD cavities, which makes it possible for the clinician to choose between the available materials. However, in the study of Sáry et al., deep but non endodontically treated cavities were tested. Root canal treatment, as it increases the depth of the cavity and further reduces coronal tooth structure, could alter the results in case of direct restorative techniques. Undoubtedly, the proper adhesive treatment of such cavities is mandatory before utilizing the splinting techniques dealt in this review. Further studies are needed to clarify the exact cavity dimensions among deep MOD cavities when indicating certain horizontal splinting techniques.

### 4.2. Fracture Pattern

Teeth with deep MOD cavities are prone to fracture, mainly resulting from the high amount of lost tooth material, and also from losing both marginal ridges [1,2]. Composite materials, due to their significantly lower fracture toughness compared to dentine, are unable to stop crack propagation [9,15,46]. Fiber reinforcement should not only be used to enhance the mechanical properties and resistance of restorations but should also deflect or possibly even stop crack propagation inside the restoration [35]. As the fracture pattern will determine the restorability of teeth in case a fracture occurs, it is of high importance. The included studies reported contradictory results regarding fracture patterns.

Five articles (Bahari et al., Daher et al., Akham et al., Küçük et al. and Mergulhao et al.) concluded that the application of horizontal long fibers resulted in a higher proportion of favorable fracture patterns. Two studies (Abou-Elnaga et al. and Belli et al.) did not give real information on whether the fractures were favorable or not in the case of using horizontal splinting techniques, and four studies (Sáry et al., Bromberg et al., Karzoun et al. and Scotti et al.) showed predominantly unfavorable fractures with horizontal splinting restorations. This is not surprising as, whenever long fibers were used for the horizontal splinting of these deep and destructed cavities, a conventional non fiber-reinforced composite was used to restore the remaining cavity. As discussed above, the conventional composite is lacking adequate fracture toughness, meaning that, if a crack develops, it cannot and will not stop in any isotropic material (e.g., conventional composite). Scotti and colleagues showed that the main front of the fracture partially deviated once it touched the layer of fibers, following the fibers’ horizontal direction. However, this effect was not sufficient to avoid a catastrophic break: the charged wall always deflected until fracture, resulting in an unfavorable fracture pattern [30]. One should consider substituting the missing dentine with SFRC in these cases in order to create a stress-absorbing layer and possibly stop crack propagation [15]. Studies are needed to explore this area.

## 5. Conclusions

Within the limitations of this review, evidence from in vitro studies suggests that horizontal long fiber placement in the form of horizontal splinting improves the fracture resistance of teeth with large MOD cavities, compared to conventional direct composite restorations. Horizontal splinting requires the usage of long fibers in a way that does not allow for the flexure of remaining cavity walls. Further studies are needed to clarify whether horizontal splinting could serve as a valid and durable alternative to indirect cupsal coverage restorations to treat large MOD cavities. As direct restorations reduce chair-time and costs for the patient, their indications and true potential should be further analyzed. The impact of horizontal splinting on the fracture patterns of directly restored teeth is not entirely clarified.

## Figures and Tables

**Table 1:** Details of the included publications.

First Author	Tested Parameter	Control Group	Type of Long Fibers	Application Technique	Main Conclusion
M. Bahari [26]	Fracture strength + Fracture pattern	Positive control (sound teeth) + Negative control (unrestored teeth)	Glass fiber	FRC post through the buccal and lingual walls + Glass fiber strip in bucco-lingually oriented groove on the restorations occlusal surface	The usage of different long fibers did not alter the fracture strength of the direct restoration compared to composite fillings in endodontically treated premolars. The fracture pattern varied according to the position and type of the long fiber.
M. Y. Abou-Elnaga [28]	Fracture resistance + Fracture pattern	Sound teeth	Glass fiber	FRC post through the buccal and lingual walls	The artificial trust access utilizing a long fiber post did not improve the fracture resistance of endodontically treated molar teeth with MOD cavities.
T. Sáry [20]	Fracture resistance + Fracture pattern	Sound teeth	Polyethylene fiber	Polyethylene fiber through the buccal and lingual walls	Using polyethylene fibers incorporated into composite fillings seems to always be beneficial in terms of fracture resistance in deep vital MOD cavities, regardless of its position within the cavity or the restoration.
C. R. Bromberg [21]	Fracture strength + Fracture pattern	Sound teeth	Glass fiber	FRC posts through the buccal and lingual walls	In case of endodontically treated molars, using transfixed fiber posts in direct fillings resulted in fracture resistance values not different to indirect overlays; however, the fracture pattern was dominantly non repairable.
R. Daher [22]	Fracture strength + Fracture pattern	Sound teeth	Glass fiber	Glass fiber strip was wrapped twice around the buccal and lingual walls	Utilizing fiber-reinforcing rings around molar MOD cavities present comparable fracture strength to indirect inlays and onlays. Furthermore, it increases the percentage of repairable fractures.
S. Belli [23]	Fracture strength + Fracture pattern	Positive control (sound teeth) + Negative control (unrestored teeth)	Polyethylene fiber	Polyethylene fiber in bucco-lingually oriented groove on the restorations’ occlusal surface	Horizontal splinting with polyethylene fibers significantly increased the fracture strength of restored endodontically treated molars.
S. Akman [27]	Mean cusp movement + Fracture strength	Composite restoration	Polyethylene fiber	Polyethylene fiber in bucco-lingually oriented groove on the restorations’ occlusal surface	Regardless of position of the fibers inside the restoration, polyethylene fibers were not able to reinforce endodontically treated MOD molar cavities.
Ö. Küçük [24]	Fracture resistance + Fracture pattern	Sound teeth + Composite restoration	Glass fiber	Glass fiber strip in bucco-lingually oriented groove on the restorations’ occlusal surface	Long glass fibers in the form of a glass fiber strip were able to strengthen root-canal-treated premolar MOD cavities to the extent of sound teeth. Furthermore, all fiber materials produced repairable fracture fractures.
W. Karzoun [25]	Fracture resistance + Fracture pattern	Positive control (sound teeth) + Negative control (unrestored teeth)	Glass fiber	FRC posts through the buccal and lingual walls	Using a horizontal glass fiber post to restore endodontically treated MOD cavities increased the fracture resistance of the restoration-tooth unit significantly.
N. Scotti [30]	Fracture resistance + Fracture pattern	Positive control (sound teeth) + Negative control (unrestored teeth)	Glass fiber	FRC posts through the buccal and lingual walls + FRC posts placed mesio-distally	Insertion of long glass fibers into the direct composite restoration in root-canal-treated molar MOD cavities was able to significant increase in their fracture resistance.
V. A. Mergulhao [29]	Fracture resistance + Fracture pattern	Sound teeth	Glass fiber	FRC posts through the buccal and lingual walls	Horizontally positioned glass fiber post did not increase the fracture resistance in case of premolar MOD cavities compared to composite fillings; however, a dominance of repairable fractures could be observed when fiber post was used.

**Table 2:** Risk of bias assessment.

First Author	Control Group	Sample Size Calculation	Standardized Samples	Randomized Samples	Single Operator	Blinded Operator	Failure Mode Evaluation	Risk of Bias
M. Bahari [26]	Yes	Yes	Yes	Yes	NA	No	Yes	Medium
M. Y. Abou-Elnaga [28]	Yes	Yes	Yes	Yes	No	No	Yes	Medium
T. Sáry [20]	Yes	No	Yes	Yes	Yes	No	Yes	Medium
C. R. Bromberg [21]	Yes	No	Yes	Yes	No	No	Yes	Medium
R. Daher [22]	Yes	No	Yes	Yes	No	No	Yes	Medium
S. Belli [23]	Yes	No	Yes	Yes	No	No	Yes	Medium
S. Akman [27]	Yes	No	Yes	Yes	NA	No	Yes	Medium
Ö. Küçük [24]	Yes	Yes	Yes	No	NA	No	Yes	Medium
W. Karzoun [25]	Yes	No	Yes	Yes	Yes	No	Yes	Medium
N. Scotti [30]	Yes	No	Yes	Yes	Yes	No	Yes	Medium
V. A. Mergulhao [29]	Yes	No	Yes	Yes	Yes	No	Yes	Medium

NA: not applicable.

**Table 3:** Mechanical testing performed within the articles.

First Author	Direction of Loading	Statical Loading	Dynamic Loading	Any Additional Tests	Investigation of Fracture Pattern
M. Bahari [26]	Vertical (long axis)	0.5 mm/min	No	No	Yes
M. Y. Abou-Elnaga [28]	Vertical (long axis)	1 mm/min	No	No	Insufficient data
T. Sáry [20]	Vertical (long axis)	2 mm/min	No	No	Yes
C. R. Bromberg [21]	Vertical (long axis)	1 mm/min	Yes (200 N, 500,000 cycles)	No	Yes
R. Daher [22]	Vertical (long axis)	1 mm/min	Yes (49 N, 600,000 cycles)	Cyclic thermal loading	Yes
S. Belli [23]	Vertical (long axis)	0.5 mm/min	No	No	Insufficient data
S. Akman [27]	Vertical (long axis)	5 mm/min	No	Cusp movement under loading	Yes
Ö. Küçük [24]	Vertical (long axis)	1 mm/min	No	No	Yes
W. Karzoun [25]	Vertical (long axis)	NA	No	No	Yes
N. Scotti [30]	45° Oblique	0.5 mm/min	Yes (50 N, 20,000 cycles)	Cyclic thermal loading	Yes
V. A. Mergulhao [29]	Vertical (long axis)	1 mm/min	Yes (0–100 N, 50,000 cycles)	Cyclic thermal loading	Yes

NA: not applicable.

## Data Availability

Not applicable.

## References

[1] Schwartz R., Robbins J. (2004). Post Placement and Restoration of Endodontically Treated Teeth: A Literature Review. J. Endod..

[2] Plotino G., Buono L., Grande N.M., Lamorgese V., Somma F. (2008). Fracture resistance of endodontically treated molars restored with extensive composite resin restorations. J. Prosthet. Dent..

[3] Maslamani M., Khalaf M., Mitra A. (2017). Association of Quality of Coronal Filling with the Outcome of Endodontic Treatment: A Follow-up Study. Dent. J..

[4] Zicari F., de Munck J., Scotti R., Naert I., van Meerbeek B. (2012). Factors affecting the cement–post interface. Dent. Mater..

[5] Soares P.V., Santos-Filho P.C.F., Martins L.R.M., Soares C.J. (2008). Influence of restorative technique on the biomechanical behavior of endodontically treated maxillary premolars. Part I: Fracture resistance and fracture mode. J. Prosthet. Dent..

[6] Scotti N., Scansetti M., Rota R., Pera F., Pasqualini D., Berutti E. (2011). The effect of the post length and cusp coverage on the cycling and static load of endodontically treated maxillary premolars. Clin. Oral Investig..

[7] Zicari F., van Meerbeek B., Scotti R., Naert I. (2012). Effect of fibre post length and adhesive strategy on fracture resistance of endodontically treated teeth after fatigue loading. J. Dent..

[8] Aurélio I.L., Fraga S., Rippe M.P., Valandro L.F. (2016). Are posts necessary for the restoration of root filled teeth with limited tissue loss? A structured review of laboratory and clinical studies. Int. Endod. J..

[9] Deliperi S., Alleman D., Rudo D. (2017). Stress-reduced Direct Composites for the Restoration of Structurally Compromised Teeth: Fiber Design According to the ‘Wallpapering’ Technique. Oper. Dent..

[10] Braga R., Boaro L., Kuroe T., Azevedo C., Singer J. (2006). Influence of cavity dimensions and their derivatives (volume and ‘C’ factor) on shrinkage stress development and microleakage of composite restorations. Dent. Mater..

[11] Garoushi S., Vallittu P., Watts D., Lassila L. (2008). Polymerization shrinkage of experimental short glass fiber-reinforced composite with semi-inter penetrating polymer network matrix. Dent. Mater..

[12] Xie K.X., Wang X.Y., Gao X.J., Yuan C.Y., Li J.X., Chu C.H. (2012). Fracture resistance of root filled premolar teeth restored with direct composite resin with or without cusp coverage: Fracture resistance of root filled premolar. Int. Endod. J..

[13] Scotti N., Eruli C., Comba A., Paolino D.S., Alovisi M., Pasqualini D., Berutti E. (2015). Longevity of class 2 direct restorations in root-filled teeth: A retrospective clinical study. J. Dent..

[14] Mangoush E., Garoushi S., Lassila L., Vallittu P.K., Säilynoja E. (2021). Effect of Fiber Reinforcement Type on the Performance of Large Posterior Restorations: A Review of In Vitro Studies. Polymers.

[15] Garoushi S., Gargoum A., Vallittu P.K., Lassila L. (2018). Short fiber-reinforced composite restorations: A review of the current literature. J. Investig. Clin. Dent..

[16] Rudo D.N., Karbhari V.M. (1999). Physical behaviors of fiber reinforcement as applied to tooth stabilization. Dent. Clin. N. Am..

[17] Belli S., Erdemir A., Ozcopur M., Eskitascioglu G. (2005). The effect of fibre insertion on fracture resistance of root filled molar teeth with MOD preparations restored with composite. Int. Endod. J..

[18] Menzies D. (2011). Systematic reviews and meta-analyses. Int. J. Tuberc. Lung Dis..

[19] Zarow M., Dominiak M., Szczeklik K., Hardan L., Bourgi R., Cuevas-Suárez C.E., Zamarripa-Calderón J.E., Kharouf N., Filtchev D. (2021). Effect of Composite Core Materials on Fracture Resistance of Endodontically Treated Teeth: A Systematic Review and Meta-Analysis of In Vitro Studies. Polymers.

[20] Sáry T., Garoushi S., Braunitzer G., Alleman D., Volom A., Fráter M. (2019). Fracture behaviour of MOD restorations reinforced by various fibre-reinforced techniques—An in vitro study. J. Mech. Behav. Biomed. Mater..

[21] Bromberg C.R., Alves C.B., Stona D., Spohr A.M., Rodrigues-Junior S.A., Melara R., Burnett L.H. (2016). Fracture resistance of endodontically treated molars restored with horizontal fiberglass posts or indirect techniques. J. Am. Dent. Assoc..

[22] Daher R., Ardu S., di Bella E., Rocca G.T., Feilzer A.J., Krejci I. (2021). Fracture strength of non-invasively reinforced MOD cavities on endodontically treated teeth. Odontology.

[23] Belli S., Erdemir A., Yildirim C. (2006). Reinforcement effect of polyethylene fibre in root-filled teeth: Comparison of two restoration techniques. Int. Endod. J..

[24] Küçük Ö., Keçeci A.D. (2021). Strengthening effect of different fiber placement designs on root canal treated and bleached premolars. Odontology.

[25] Karzoun W., Abdulkarim A., Samran A., Kern M. (2015). Fracture Strength of Endodontically Treated Maxillary Premolars Supported by a Horizontal Glass Fiber Post: An In Vitro Study. J. Endod..

[26] Bahari M., Mohammadi N., Kimyai S., Kahnamoui M.A., Vahedpour H., Torkani MA M., Oskoee A.S. (2019). Effect of Different Fiber Reinforcement Strategies on the Fracture Strength of Composite Resin Restored Endodontically Treated Premolars. Pesqui. Bras. Odontopediatr. Clín. Integr..

[27] Akman S., Akman M., Eskitascioglu G., Belli S. (2011). Influence of several fibre-reinforced composite restoration techniques on cusp movement and fracture strength of molar teeth: Effect of fibre-reinforced restorations on cusp movement. Int. Endod. J..

[28] Abou-Elnaga M.Y., Alkhawas M.-B.A.M., Kim C.H., Refai A.S. (2019). Effect of Truss Access and Artificial Truss Restoration on the Fracture Resistance of Endodontically Treated Mandibular First Molars. J. Endod..

[29] Mergulhão V., de Mendonça L., de Albuquerque M., Braz R. (2019). Fracture Resistance of Endodontically Treated Maxillary Premolars Restored with Different Methods. Oper. Dent..

[30] Scotti N., Forniglia A., Tempesta R.M., Comba A., Saratti C.M., Pasqualini D., Alovisi M., Berutti E. (2016). Effects of fiber-glass-reinforced composite restorations on fracture resistance and failure mode of endodontically treated molars. J. Dent..

[31] Chai H., Lawn B.R. (2017). Fracture resistance of molar teeth with mesial-occlusal-distal (MOD) restorations. Dent. Mater..

[32] Alleman D., Alleman D., Deliperi S., Diaz J.A., Martins L., Keulemans F. (2021). Decoupling with Time. Inside Dentistry.

[33] Lassila L., Keulemans F., Säilynoja E., Vallittu P.K., Garoushi S. (2018). Mechanical properties and fracture behavior of flowable fiber reinforced composite restorations. Dent. Mater..

[34] Başaran E.G., Ayna E., Vallittu P.K., Lassila L.V.J. (2013). Load bearing capacity of fiber-reinforced and unreinforced composite resin CAD/CAM-fabricated fixed dental prostheses. J. Prosthet. Dent..

[35] Garoushi S., Vallittu P.K., Lassila L.V.J. (2007). Short glass fiber reinforced restorative composite resin with semi-inter penetrating polymer network matrix. Dent. Mater..

[36] Khan S.I.R., Ramachandran A., Alfadley A., Baskaradoss J.K. (2018). Ex vivo fracture resistance of teeth restored with glass and fiber reinforced composite resin. J. Mech. Behav. Biomed. Mater..

[37] Kim S.G., Kim S.S., Levine J.L., Piracha Y.S., Solomon C.S. (2020). A Novel Approach to Fracture Resistance Using Horizontal Posts after Endodontic Therapy: A Case Report and Review of Literature. J. Endod..

[38] Scotti N., Coero Borga F.A., Alovisi M., Rota R., Pasqualini D., Berutti E. (2012). Is fracture resistance of endodontically treated mandibular molars restored with indirect onlay composite restorations influenced by fibre post insertion?. J. Dent..

[39] Szabó P.B., Sáry T., Szabó B. (2019). The key elements of conducting load-to-fracture mechanical testing on restoration-tooth units in restorative dentistry. Analecta Tech. Szeged..

[40] Tang W., Wu Y., Smales R.J. (2010). Identifying and Reducing Risks for Potential Fractures in Endodontically Treated Teeth. J. Endod..

[41] Ozsevik A.S., Yildirim C., Aydin U., Culha E., Surmelioglu D. (2016). Effect of fibre-reinforced composite on the fracture resistance of endodontically treated teeth: Coronal Strength of Root-Filled Teeth. Aust. Endod. J..

[42] Wu Y., Cathro P., Marino V. (2010). Fracture resistance and pattern of the upper premolars with obturated canals and restored endodontic occlusal access cavities. J. Biomed. Res..

[43] Taha N.A., Palamara J.E.A., Messer H.H. (2009). Cuspal deflection, strain and microleakage of endodontically treated premolar teeth restored with direct resin composites. J. Dent..

[44] Hood J.A. (1991). Biomechanics of the intact, prepared and restored tooth: Some clinical implications. Int. Dent. J..

[45] Forster A., Braunitzer G., Tóth M., Szabó B.P., Fráter M. (2019). In Vitro Fracture Resistance of Adhesively Restored Molar Teeth with Different MOD Cavity Dimensions: Fracture Resistance of Adhesively Restored MOD Cavities. J. Prosthodont..

[46] Lassila L., Säilynoja E., Prinssi R., Vallittu P.K., Garoushi S. (2020). Fracture behavior of Bi-structure fiber-reinforced composite restorations. J. Mech. Behav. Biomed. Mater..

